# Exposure to the anti-microbial chemical triclosan disrupts keratinocyte function and skin integrity in a model of reconstructed human epidermis

**DOI:** 10.1080/1547691X.2022.2148781

**Published:** 2023-12

**Authors:** Rachel Baur, Michael Kashon, Ewa Lukomska, Lisa M. Weatherly, Hillary L. Shane, Stacey E. Anderson

**Affiliations:** a Allergy and Clinical Immunology Branch, Health Effects Laboratory Division, National Institute for Occupational Safety and Health, Morgantown, WV, USA; b Department of Microbiology, Immunology, and Cell Biology, School of Medicine, West Virginia University, Morgantown, WV, USA

**Keywords:** Triclosan, keratinocytes, skin, barrier function, permeability

## Abstract

Triclosan is an anti-microbial chemical incorporated into products that are applied to the skin of healthcare workers. Exposure to triclosan has previously been shown to be associated with allergic disease in humans and impact the immune responses in animal models. Additionally, studies have shown that exposure to triclosan dermally activates the NLRP3 inflammasome and disrupts the skin barrier integrity in mice. The skin is the largest organ of the body and plays an important role as a physical barrier and regulator of the immune system. Alterations in the barrier and immune regulatory functions of the skin have been demonstrated to increase the risk of sensitization and development of allergic disease. In this study, the impact of triclosan exposure on the skin barrier and keratinocyte function was investigated using a model of reconstructed human epidermis. The apical surface of reconstructed human epidermis was exposed to triclosan (0.05–0.2%) once for 6, 24, or 48 h or daily for 5 consecutive days. Exposure to triclosan increased epidermal permeability and altered the expression of genes involved in formation of the skin barrier. Additionally, exposure to triclosan altered the expression patterns of several cytokines and growth factors. Together, these results suggest that exposure to triclosan impacts skin barrier integrity and function of human keratinocytes and suggests that these alterations may impact immune regulation.

## Introduction

Triclosan is an anti-microbial chemical that has been used in healthcare settings and in consumer products such as soaps, deodorants, toothpastes, and mouthwashes, since 1972 (Jones et al. 2000; Fang et al. 2010). Although the use of triclosan has been banned from consumer soaps by the US Food and Drug Administration (FDA (Food and Drug Administration) 2016), triclosan is still used in certain formulations of products used in healthcare settings (Consumer product information database). Exposure to triclosan has been associated with disease, including allergic disease (reviewed in Weatherly and Gosse 2017; Anderson et al. 2019). Results from the 2005–2006 National Health and Nutrition Examination Survey (NHANES) show that exposure to triclosan is positively associated with food sensitization and aeroallergy (Savage et al. 2012). Additionally, results from the 2005–2010 NHANES demonstrate that exposure to triclosan is positively associated with asthma exacerbation (Savage et al. 2014). In mice, although triclosan has not been shown to be a sensitizer (Anderson et al. 2016), exposure to triclosan on the skin has been demonstrated to augment the allergic response in an asthma model (Anderson et al. 2013) and in a mouse model of peanut allergy (Tobar et al. 2016). Additionally, oral exposure to triclosan has been shown to increase the airway response to house dust mites in mice (Hirota et al. 2019).

Exposure to triclosan can occur dermally or orally, due to the use of skin and oral care products containing triclosan. Health care workers using triclosan-containing soap have been shown to have higher levels of urinary triclosan, demonstrating that triclosan is absorbed through the skin (MacIsaac et al. 2014). Furthermore, animal studies have shown that triclosan is absorbed through mouse skin and excreted through feces and urine (Fang et al. 2016). Together these studies show that triclosan can be absorbed through the skin, passed through tissues in the body, and excreted from the body. Studies also suggest that triclosan can impact the skin microenvironment. Exposure to triclosan on cultured human keratinocytes has been shown to disrupt metabolic pathways and lipid levels, increase production of reactive oxygen species, and increase pro-inflammatory cytokines (interleukin [IL]-1b, IL-6, IL-8, tumor necrosis factor [TNF]-a) (Liang et al. 2021). In mice, topical triclosan exposure has been shown to increase transepidermal water loss, a measure of disrupted skin barrier integrity, and alter the expression of genes involved in the skin barrier (Baur et al. 2021). Together, these results suggest that dermal exposure to triclosan alters immune responses and that this may be influenced by mediators such as skin barrier integrity.

The skin, comprised of the epidermis and dermis, is the first line of defense against environmental exposures and influences immune responses. Distinct features of the physical skin barrier, such as lipids, cornified envelopes in the stratum corneum, and tight junctions in the stratum granulosum, limit or prevent the passage of certain molecules, such as high molecular weight proteins, through the skin (Goleva et al. 2019). When barrier integrity or permeability are disrupted, chemicals and/or proteins are more likely to be absorbed through the skin (Rietz Liljedahl et al. 2021), and may lead to increased allergic sensitization. Additionally, in children, elevated levels of transepidermal water loss or mutations in the skin barrier gene filaggrin are a risk factor for development of atopic dermatitis or food allergy (Kelleher et al. 2015, 2016). Due to the use of triclosan in skin products, the skin is a primary route of exposure through which triclosan can be absorbed (MacIsaac et al. 2014). Thus, investigating the skin barrier integrity and response of keratinocytes, the most abundant cell type in the epidermis, to triclosan exposure is critical to understanding the effects on the immune system and associations with increased sensitization potential.

Keratinocytes are key players in the structural integrity of the skin and they are involved in sensing and reacting to the environment (Hammad and Lambrecht 2015). Cytokine signaling is a key mechanism that keratinocytes use to interact with immune cells, both by producing cyto-kines and responding to cytokines *via* cytokine receptors (Hammad and Lambrecht 2015; Jiang et al. 2020). Certain cytokines that are produced by epithelial cells including keratinocytes, such as thymic stromal lymphopoietin (TSLP), IL-25, and IL-33, can communicate with immune cells and skew immune responses toward T-helper cell Type 2 (T_H_2) responses, leading to increased risk of sensitization (Hammad and Lambrecht 2015; Goleva et al. 2019). Dermal exposure to triclosan has been shown to increase levels of *Tslp*, as well as other signaling molecules such as S100 calcium-binding protein A8 (*S100a8*), IL-1β (*Il1b*), chemokine (C-X-C motif) ligand (*Cxcl*)1, and *Cxcl2* in mouse skin (Anderson et al. 2020; Weatherly et al. 2020). Additionally, triclosan exposure on mouse skin has been shown to alter expression of filaggrin and keratin genes, demonstrating an impact on keratinocytes (Baur et al. 2021). Exposure to triclosan on human keratinocytes *in vitro* has been shown to alter metabolic pathways and increase pro-inflammatory cytokines (Liang et al. 2021). However, the impact of triclosan exposure on human keratinocyte response and the skin barrier is not well understood.

Three-dimensional (3D) skin models have been successfully employed to evaluate the skin barrier and to explore mechanisms of skin disease (Niehues et al. 2018). One type of 3D skin model, i.e. reconstructed human epidermis (RHE), represents a particularly useful approach to explore the skin barrier and keratinocyte function independent from immune cells and the microbiome. RHE is comprised of a functional barrier with an intact stratum corneum, proliferating and differentiating primary human keratinocytes, and has similarities in the multi-layered structure of intact human skin (Frankart et al. 2012). In this study, the impact of triclosan exposure on RHE barrier and keratinocyte response was evaluated by assessing barrier permeability and expression of barrier genes, cytokines, and growth factors.

## Materials and methods

### RHE tissue

EpiDerm^™^ (MatTek, Ashland, MA) tissues were equilibrated as per manufacturer instructions. In brief, tissue inserts were placed at 4 °C for 2 hr upon arrival, then transferred to 6-well plates containing 0.9 ml/well of pre-warmed Dulbecco’s Modified Eagle’s Medium (DMEM, hydrocortisone-free) containing epidermal growth factor, insulin, other proprietary stimulators of epidermal differentiation, gentamicin (5 μg/ml), amphotericin B (0.25 μg/ml), phenol red, and proprietary lipid precursors (MatTek). Tissues were incubated at 37 °C with 5% CO_2_ overnight to equilibrate. The following morning, tissues were washed with 100 μl pre-warmed Dulbecco’s phosphate-buffered saline (DPBS) and either (1) media was replaced with 0.9 ml fresh pre-warmed DMEM media/well (5-day experiments) or (2) tissues were transferred to 12-well hanging-top lids (MatTek) with plates containing 5 ml pre-warmed DMEM media per well (6, 24, and 48 h experiments). For the 5-day experiment, media was replaced daily throughout the experiment, prior to each exposure.

### Triclosan exposures

Triclosan (CAS #3380–34-5) was purchased from EMD Millipore Corp. (Burlington, MA, USA). Acetone was selected as the vehicle based on solubility and previous use in evaluating triclosan exposure on EpiDerm tissues (Marshall et al. 2015). Acetone (CAS #67–41-1) was purchased from Sigma (St. Louis, MO, USA). EpiDerm tissues (*n =* 3/group) were exposed on the apical side to 30 μl acetone (vehicle) or triclosan (0.05–0.2%) dissolved in acetone (*w/v*) once for 6, 24, or 48 h or once/day (30 μl/day) for 5 consecutive days. Experiments were independently performed twice for each timepoint and endpoint. An additional experiment was performed to compare no exposure versus acetone control. Tissues were incubated at 37 °C in 5% CO_2_ during the exposure. The concentrations were selected based on triclosan concentrations in product formu-lations (0.1–0.3%) and a previous toxicity assessment of EpiDerm tissues to triclosan where levels of ≥0.188% triclosan were determined to be toxic (Marshall et al. 2015).

### LDH release assay

Culture media (10 μL) was collected after 6, 24, or 48 h of the single exposure or each day of the 5-day exposure and transferred to a clear flat-bottomed 96-well plate. The LDH-Cytotoxicity Colorimetric Assay Kit II (BioVision, Milpitas, CA, USA) was used to perform the lactate dehydrogenase (LDH) release assay per manufacturer instructions. Absorbance (at 450 nm) was measured in duplicate for each sample using a mQuant (BioTek, Winooski, VT, USA) or Varioskan Lux (Thermo Scientific, Waltham, MA, USA) microplate spectrophotometer. Values were averaged and fold-changes compared to vehicle control were calculated.

### Gene expression analysis

EpiDerm tissues were washed with 100 μl pre-warmed DPBS immediately prior to nucleic acid extraction. EpiDerm tissues were disrupted and homogenized in 700 μl QIAzol (Qiagen, Hilden, Germany) using a steel bead and TissueLyser II or with a pellet pestle homo-genizer. Homogenates were centrifuged at 15,000 ×g for 10 min at 4 °C. Total RNA was isolated from lysates using the miRNAeasy kit (Qiagen) per manufacturer instructions, with a final elution volume of 30 μl per sample. RNA purity and yield were determined on a NanoDrop Spectrophotometer (Thermo Scientific). Reverse transcription was performed using the High-Capacity cDNA Reverse Transcription Kit (Applied Biosystems, Waltham, MA, USA) according to manufacturer instructions. TaqMan Fast Universal PCR Master Mix (Applied Biosystems), cDNA, and gene-specific primers (TaqMan Gene Expression Assays) were combined, and real-time quantitative PCR was performed per manufacturer’s instructions. Gene expression was analyzed on a 7500 Fast Real-Time PCR System, StepOnePlus, or QuantStudio3 (all Applied Biosystems) system using cycling conditions recommended by the manufacturer. *GAPDH* (Hs02786624_g1) was used as the reference gene. Data was collected and relative fold change compared to the acetone vehicle was calculated using the cycle threshold (C_t_) and the 2^−ΔΔCt^ method (Livak and Schmittgen 2001). Data from one tissue insert (5-day exposure) was identified to be an outlier (using a Grubbs’ test) and so was excluded from the analysis.

Genes evaluated include filaggrin (*FLG*) (Hs00856927_g1), *FLG2* (Hs00418578_m1), involucrin (*IVL*) (Hs00846307_s1), loricrin (*LOR*) (Hs01894962_s1), tight junction protein 1 (*TJP1*) (Hs01551861_m1), occludin (*OCLN*) (Hs00170162_m1), keratin (*KRT*) 10 (*KRT10*) (Hs00166289_m1), *KRT14* (Hs00265033_m1), e-cadherin (*CDH1*) (Hs01023895_m1), *TSLP* (Hs00263639_m1), *S100A8* (Hs00374264_g1), *IL1A* (Hs00174092_m1), *IL1B* (Hs01555410_m1), tumor necrosis factor (*TNF*) (Hs00174128_m1), *CXCL1* (Hs00236937_m1), *CXCL2* (Hs00601975_m1), and *CXCL8* (Hs00174103_m1).

### Cytokine release

Culture media was collected and frozen at −80 °C. Media was evaluated with a custom human premixed multi-analyte kit (LXSAHM; R&D Systems, Minneapolis, MN, USA) and analyzed on a MagPix (Luminex) following manufacturer protocols. Analytes measured included: epidermal growth factor (EGF), interleukin (IL)-1α, IL-6, IL-18, IL-33, S100A8, tumor necrosis factor (TNF)-α, vascular endothelial growth factor (VEGF), granulocyte macrophage colony-stimulating factor (GM-CSF), IL-1β, IL-8, IL-31, IL-36β, transforming growth factor (TGF)-α, and thymic stromal lymphopoietin (TSLP). Levels of sensitivity for each analyte are available at the R&D Systems website (https://www.rndsystems.com/products/human-luminex-discovery-assay_lxsahm).

### Permeability assay

EpiDerm tissues were washed with 100 μl pre-warmed DPBS immediately prior to the permeability assay. Tissues were exposed on the apical side to 40 μl of 1 mM Lucifer Yellow CH dilithium salt (Sigma) dissolved in DPBS and incubated at 37 °C in 5% CO_2_ for 2 h. Media (100 μl/replicate) was collected and fluorescence intensity was measured in triplicate in 96-well black flat-bottomed plates (Greiner Bio-One, Monroe, NC) read on a Synergy (BioTek) or a Varioskan Lux microplate reader (excitation 428 nm, emission 536 nm). Fluorescence intensity was averaged per sample and fold-change in fluorescence intensity compared to vehicle control was calculated.

### Histology

Following the permeability assay, EpiDerm tissues were washed with 100 μl pre-warmed DPBS immediately prior to collection. Formalin-fixed paraffin-embedded tissues were sectioned (5 μm) and mounted on slides and stained with hematoxylin and eosin following standard procedures (1 slide/tissue). Slides were brightfield imaged on an VS120 slide scanner or AX70 microscope (both Olympus, Tokyo, Japan). Epidermal thickness was measured from three random views/slide and three measurements/view were taken and averaged.

### Statistical analysis

The three replicate samples from two independent experiments were averaged for each treatment combination resulting in a sample size of two/group. Dependent measures were analyzed using mixed-model analysis of variance (ANOVA), with each analysis incorporating experiment as a random factor. A two-way factorial ANOVA with repeated measures was utilized when time and exposure were included as independent variables and repeated measures were a factor. A simple two-way factorial ANOVA was used when time and exposure were included as independent variables. A one-way ANOVA was utilized when only exposure was included as an independent variable. In some cases, data were log-transformed to reduce heterogeneous variance and meet the assumptions of the analysis. *Post-hoc* comparisons were carried out using a Fishers LSD test. All differences were considered significant at *p* < 0.05. All analyses were carried out using SAS version 9.4 software (SAS Institute, Cary, NC, USA).

## Results

### Exposure to triclosan altered expression of barrier genes

**Figure 1.**
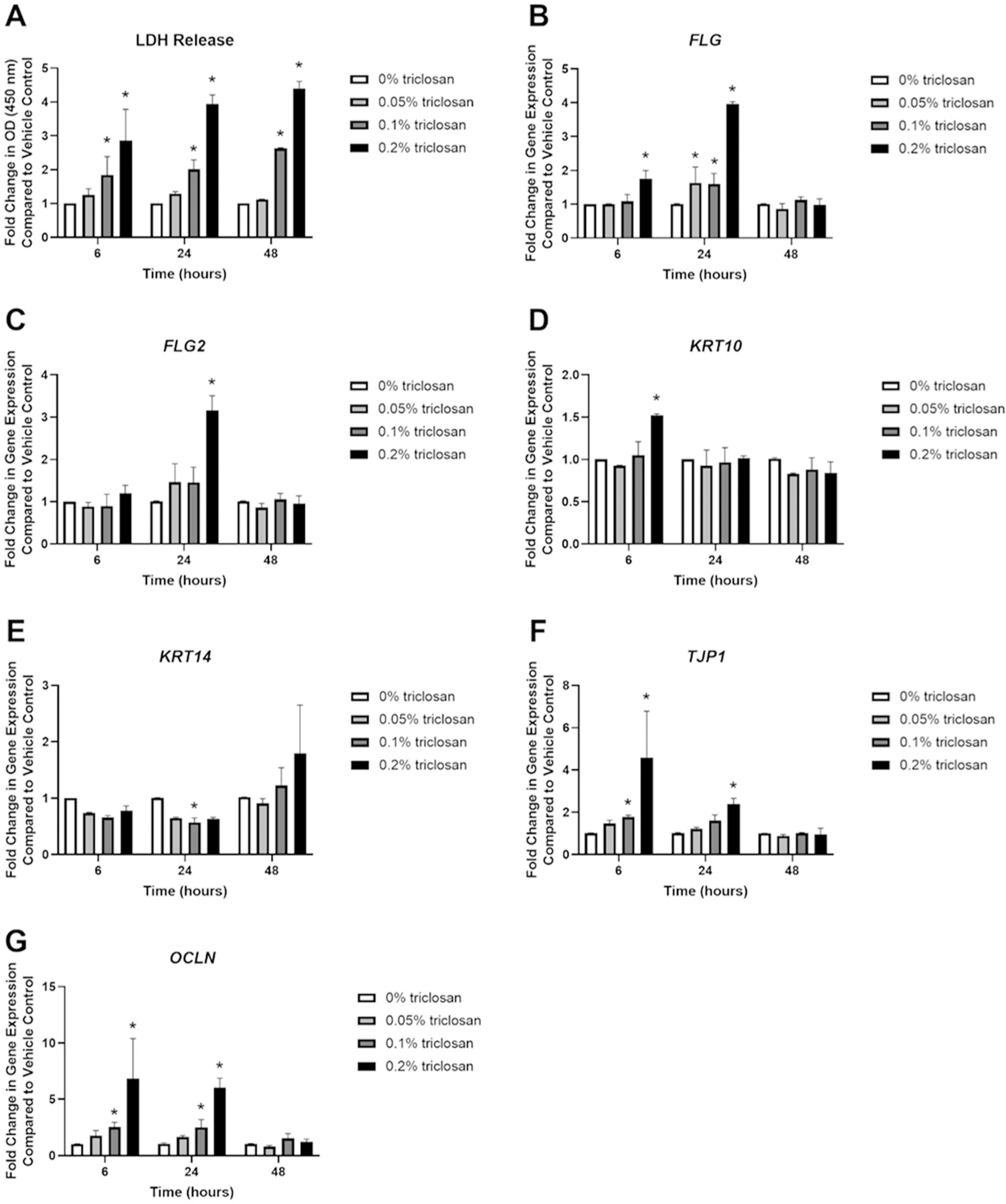
Exposure to triclosan induced toxicity and altered expression of barrier genes in EpiDerm tissues. (A) Fold-change in optical density (OD) (450 nm) compared to vehicle control in culture media to detect LDH release following 6, 24, and 48 h of 0% triclosan (acetone vehicle) or 0.05–0.2% triclosan. Bars represent mean (± SEM) of two samples/group. *p < 0.05 vs. 0% triclosan. Fold-change in gene expression compared to vehicle control of (B) *FLG*, (C) *FLG2*, (D) *KRT10*, (E) *KRT14*, (F) *TJP1*, and (G) *OCLN* following 6, 24, and 48 hr of 0% triclosan (vehicle) or 0.05–0.2% triclosan. Bars represent mean (± SEM) of two samples/group. *p < 0.05 vs. 0% triclosan.

Single exposure to triclosan for 6 (0.1 and 0.2% triclosan), 24 (0.1 and 0.2% triclosan), and 48 (0.1% and 0.2% triclosan) hr increased LDH levels, a marker of cytotoxicity, in culture media in a dose dependent manner (Figure 1(A)). Additionally, RNA yield from the tissues was reduced following 6 and 24 h of 0.2% triclosan exposure and following 48 h of 0.1 and 0.2% triclosan exposure (Supplemental Figure 1(A)). Select genes known to be 0involved in barrier integrity (previously investigated in mice; see Baur et al. 2021) were then evaluated. Exposure to triclosan for 6 (0.2%) and 24 (0.05, 0.1, and 0.2%) h significantly increased expression of *FLG* (Figure 1(B)) and 24 h of 0.2% triclosan exposure significantly increased *FLG2* expression (Figure 1(C)). However, the increase in *FLG* and *FLG2* did not persist at the 48-h timepoint. Exposure to 0.2% triclosan for 6 h increased *KRT10* expression (Figure 1(D)). *KRT14* was decreased at 24 h (0.1%) (Figure 1(E)). Exposure to triclosan for 6 (0.1 and 0.2%) and 24 (0.2%) h increased expression of *TJP1* (Figure 1(F)) and *OCLN* (0.1 and 0.2% at 6 h, 0.1 and 0.2% at 24 h) (Figure 1(G)). Furthermore, exposure to 0.2% triclosan increased expression of *IVL*, *LOR* and *CDH1* at multiple timepoints (Supplemental Figure 1(B–D)). Together, these data demonstrate that *in vitro* triclosan exposure alters the expression of select skin barrier genes in EpiDerm.

### Exposure to triclosan increased expression of cytokines and tissue permeability

**Figure 2.**
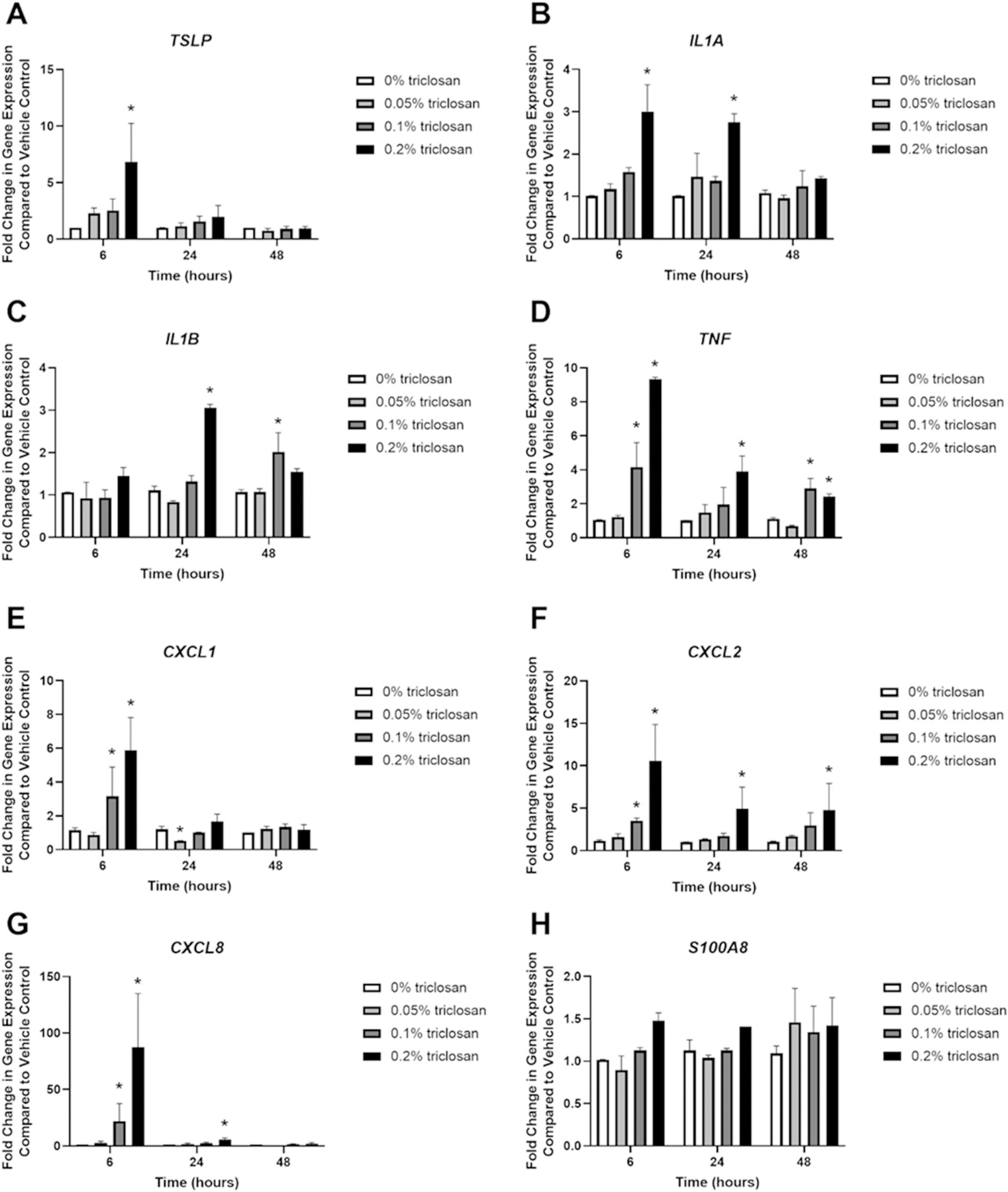
Exposure to triclosan on EpiDerm tissues altered the gene expression of cytokines. Fold-change in gene expression compared to vehicle control of (A) *TSLP*, (B) *IL1A*, (C) *IL1B*, (D) *TNF*, (E) *CXCL1*, (F) *CXCL2*, (G) *CXCL8*, and (H) *S100A8* following 6, 24, and 48 h of 0% triclosan (acetone vehicle) or 0.05–0.2% triclosan. Bars represent mean (± SEM) of two samples/group. *p < 0.05 vs. 0% triclosan.

Single exposure to 0.2% triclosan for 6 h significantly increased the gene expression of *TSLP* (Figure 2(A)) while exposure to 0.2% triclosan for 6 and 24 h significantly increased the expression of *IL1A* (Figure 2(B)). Twenty-four h of 0.2% triclosan and 48 h of 0.1% triclosan exposure significantly increased expression of *IL1B* (Figure 2(C)). Exposure to triclosan for 6 h (0.1 and 0.2%), 24 h (0.2%), and 48 h (0.1 and 0.2%) also increased *TNF* expression (Figure 2(D)). Triclosan exposure increased the expression of chemokines *CXCL1* (Figure 2(E)) (6 h, 0.1 and 0.2%), *CXCL2* (Figure 2(F)) (6 h, 0.1 and 0.2%; 24 h, 0.2%; 48 h, 0.2%), and *CXCL8* (Figure 2(G)) (6 h, 0.1 and 0.2%; 24 h, 0.2%). Exposure to 0.05% triclosan for 24 h decreased *CXCL1* expression. Exposure to triclosan had no significant impact on *S100A8* expression (Figure 2(H)).

To evaluate cytokines at the protein level, media was collected following triclosan (0.05, 0.1, 0.2%) exposure on EpiDerm tissues for 24 and 48 h. Corresponding with changes in gene expression, 24 (0.1 and 0.2%) and 48 (0.1 and 0.2%) h of triclosan exposure significantly increased release of IL-1α in the media (Table 1). In contrast, no significant changes in CXCL8 levels in the culture media were observed (Table 1). Additional cytokines and growth factors known to be produced by keratinocytes were evaluated following triclosan exposure. Exposure to 0.1% and 0.2% triclosan for 24 and 48 h significantly increased levels of IL-36 and triclosan exposure (0.05, 0.1, and 0.2%) for 24 h increased VEGF levels (Table 1). Additionally, exposure to 0.1% triclosan for 48 h increased VEGF. Exposure to triclosan did not alter levels of EGF in the culture media. TSLP, TNFα, S100A8, IL-1β, IL-18, IL-31, IL-33, GMCSF, TGFα, and IL-6 levels were near or below the limit of detection (results not shown).

**Figure 3.**
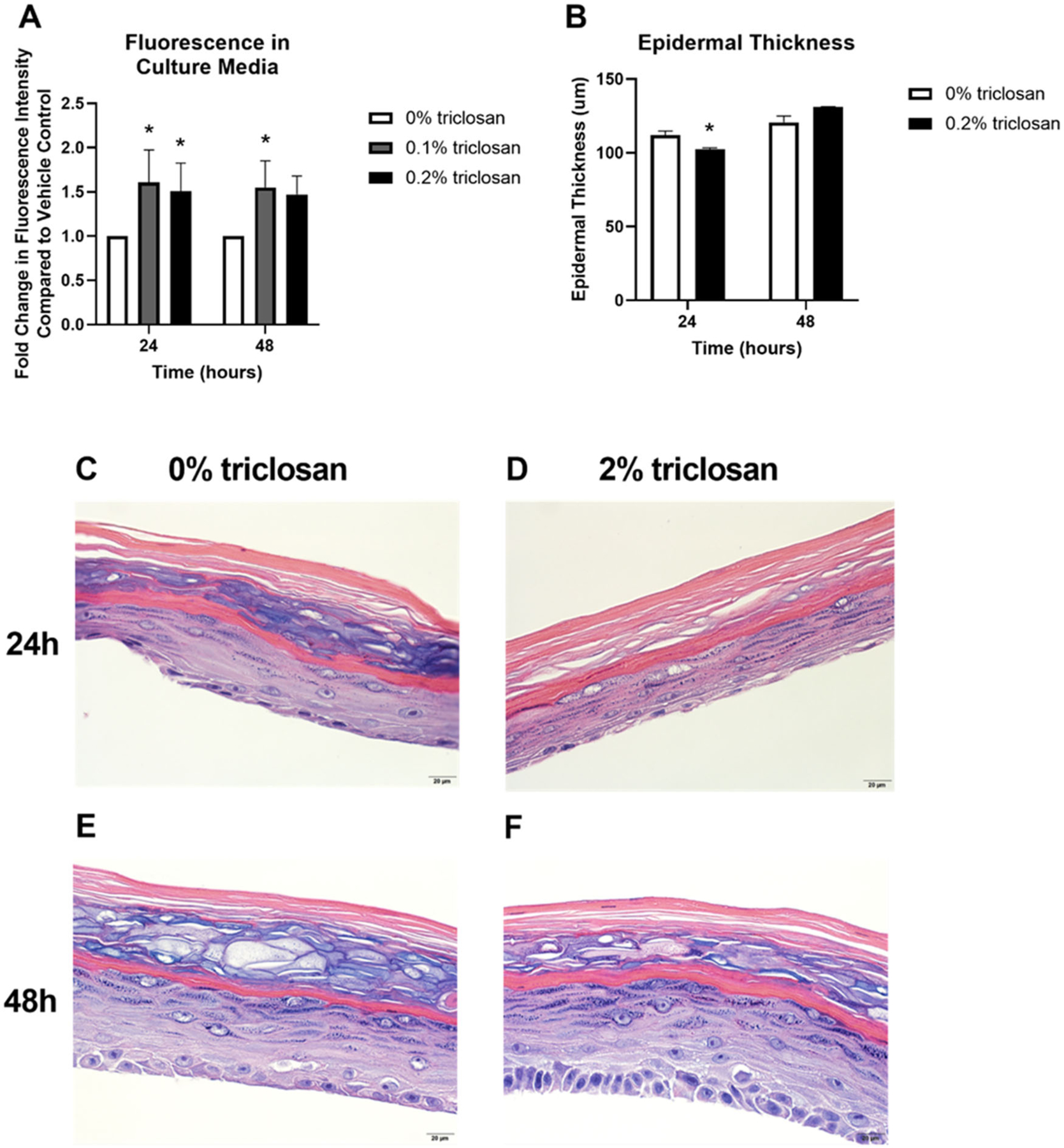
Exposure to triclosan on EpiDerm tissues altered barrier permeability. (A) Fold-change in fluorescence intensity compared to vehicle control following 24 and 48 h of 0% triclosan (vehicle) or 0.1–0.2% triclosan. Bars represent mean (± SEM) of two samples/group. *p < 0.05 vs. 0% triclosan. (B) Epidermal thickness (μm) following 24 and 48 hr of 0% triclosan (acetone vehicle) or 0.2% triclosan. Bars represent mean (± SEM) of two samples/group. *p < 0.05 vs. 0% triclosan. Representative hematoxylin and eosin images of EpiDerm tissues following 24 h of exposure to acetone vehicle (C) or 0.2% triclosan (D) and 48 h of exposure to acetone vehicle (E) or 0.2% triclosan (F). Scale bar = 20 μm.

To investigate the impact of triclosan exposure on keratinocyte function, permeability of a fluorescent molecule through the EpiDerm tissue was assessed after 24 and 48 h of exposure to the mid (0.1%) and high (0.2%) concentrations of triclosan. Exposure to triclosan on EpiDerm tissues for 24 (0.1 and 0.2%) and 48 (0.1%) h significantly increased tissue permeability (Figure 3(A)). However, epidermal thickness was decreased only at 24 h (Figure 3(B)) and minimal changes in morphology (Figure 3(C–F)) following 0.2% triclosan exposure were identified. Taken together, these results demonstrate that a single exposure to triclosan increases expression of select cytokines and growth factors in EpiDerm and increases tissue permeability.

### Repeated exposure to triclosan had minimal impacts on expression of skin barrier genes

**Figure 4.**
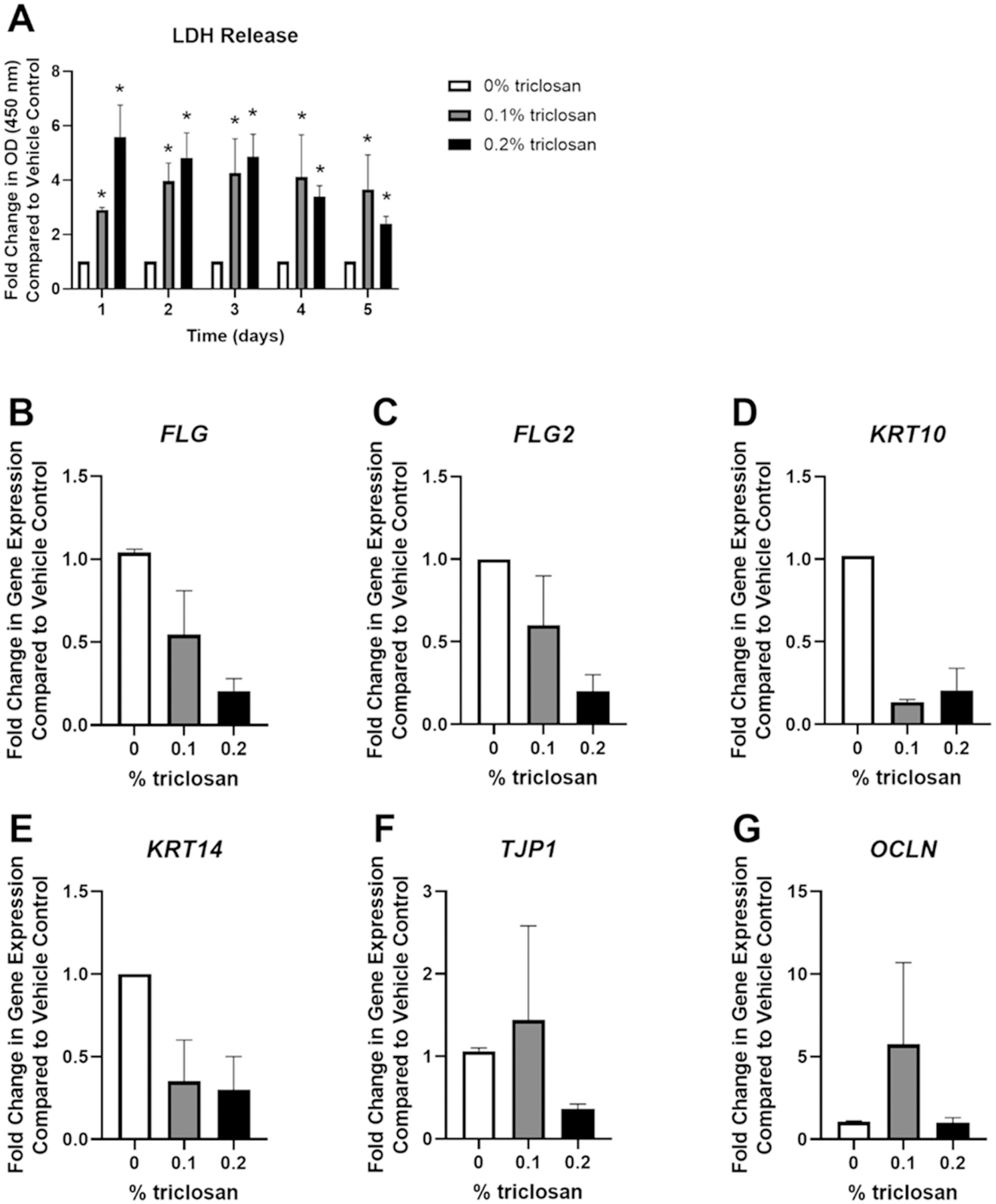
Repeated exposure to triclosan increased toxicity in EpiDerm tissues. (A) Fold-change in optical density (OD) (450 nm) compared to vehicle control in culture media to detect LDH release following 5 days of 0% triclosan (acetone vehicle) or 0.1–0.2% triclosan. Bars represent mean (± SEM) of two samples/group. *p < 0.05 vs. 0% triclosan. Fold-change in gene expression compared to vehicle control of (B) *FLG*, (C) *FLG2*, (D) *KRT10*, (E) *KRT14*, (F) *TJP1*, and (G) *OCLN* following 5 days of 0% triclosan (acetone vehicle) or 0.1–0.2% triclosan. Bars represent mean (± SEM) of two samples/group.

To evaluate the impact of repeated daily exposures to triclosan, as has previously been investigated in mice (Baur et al. 2021), EpiDerm tissues were exposed daily for 5 consecutive days to triclosan (0.1 and 0.2%). Exposure to triclosan (0.1 and 0.2%) for 1–5 days signifi-cantly increased LDH release in media (Figure 4(A)). Additionally, RNA yield was reduced after 5 days of triclosan (0.1 and 0.2%) exposure (Supplemental Figure 2(A)). Daily exposure of EpiDerm tissues to triclosan for a period of 5 days had no significant impact on the expression of *FLG*, *FLG2*, *KRT10*, *KRT14, TJP1*, or *OCLN* (Figure 4(B–G)). Additionally, no significant change in expression of *IVL* nor *CDH1* was observed following triclosan exposure, but 0.2% triclosan significantly decreased *LOR* expression (Supplementary Figure 2(B–D)). Together, these data demonstrate that repeated exposure to triclosan on EpiDerm tissues had minimal impacts on the expression of skin barrier genes.

### Repeated exposure to triclosan changed expression of cytokines and growth factors

**Figure 5.**
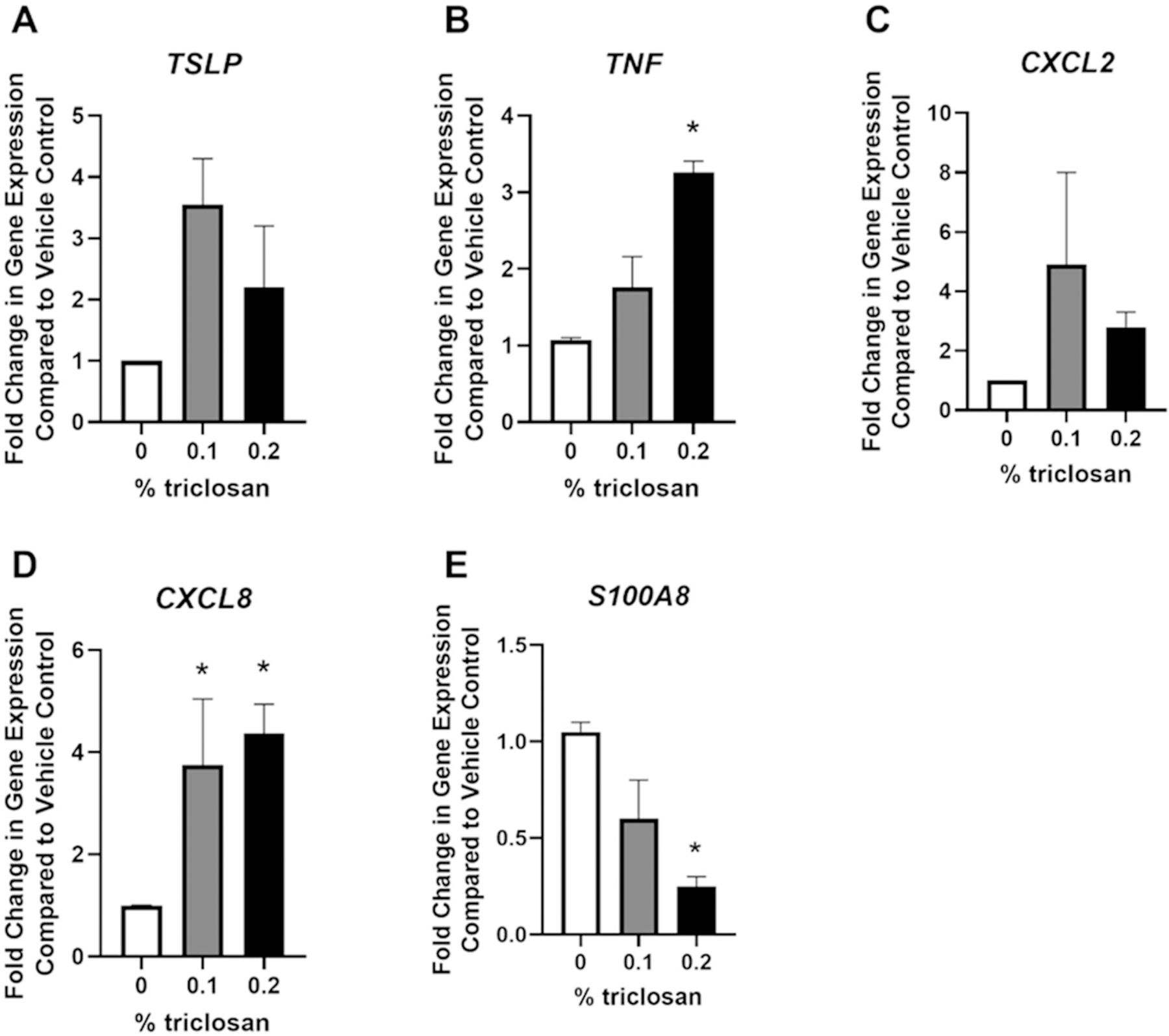
Repeated exposure to triclosan on EpiDerm tissues changed expression of immune-related genes. Fold-change in gene expression compared to vehicle control of (A) *TSLP*, (B) *TNF*, (C) *CXCL2*, (D) *CXCL8*, and (E) *S100A8* following 5 days of 0% triclosan (acetone vehicle) or 0.1–0.2% triclosan. Bars represent mean (± SEM) of two samples/group. *p < 0.05 vs. 0% triclosan.

Repeated daily exposure of EpiDerm tissues to triclosan had no significant impact on *TSLP* or *CXCL2* gene expression (Figure 5(A,C)). Exposure to triclosan for 5 days signifi-cantly increased the gene expression of *TNF* (Figure 5(B), 0.2%) and *CXCL8* (Figure 5(D), 0.1 and 0.2%). In contrast, triclosan exposure for 5 days significantly decreased gene expression of *S100A8* (Figure 5(E), 0.2%).

**Figure 6.**
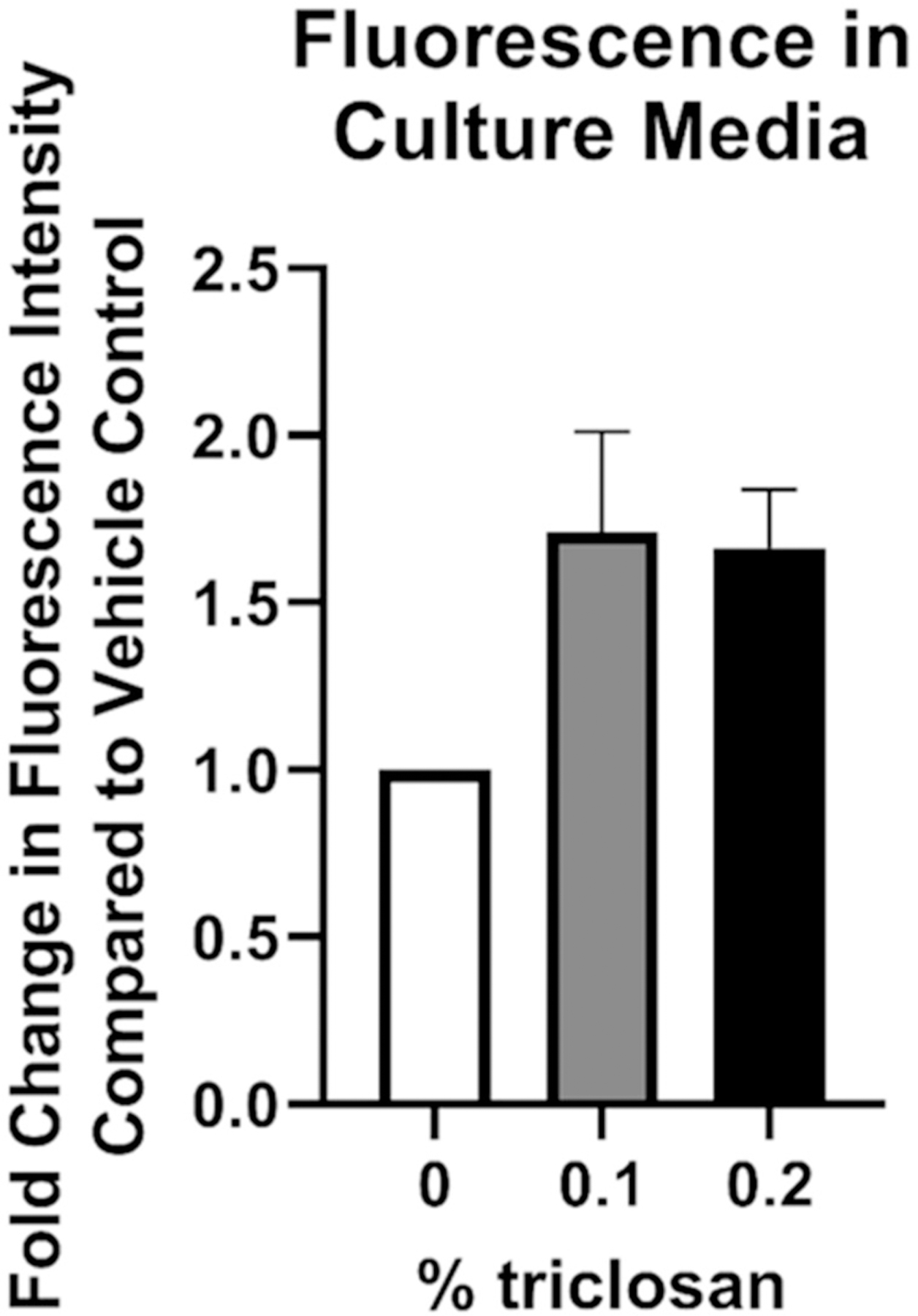
Repeated exposure to triclosan on EpiDerm tissues had no significant impact on permeability. Fold-change in fluorescence intensity compared to vehicle control following 5 days of 0% triclosan (acetone vehicle) or 0.1–0.2% triclosan. Bars represent mean (± SEM) of two samples/group.

Media was collected daily prior to each exposure, up to 5 days of exposure, to investigate changes in cytokine excretion over time. Exposure to triclosan (0.2%) increased IL-1α production as early as 1-day post exposure (Table 2), as shown previously with a single triclosan exposure (Table 1). IL-1α levels continued to increase at 2 days of triclosan exposure before plateauing at Days 3–5. Exposure to 0.2% triclosan significantly increased IL-36 through 5 days of exposure (Table 2). After repeated triclosan (0.1 and 0.2%) exposure, EGF levels were significantly increased (Table 2). In contrast, repeated exposure to triclosan decreased the levels of CXCL8 and VEGF in the culture media (Table 2). TSLP, TNFα, S100A8, IL-1β, IL-18, IL-31, IL-33, GMCSF, TGFα, and IL-6 levels were low or undetectable (results not shown). Exposure to acetone alone compared to no exposure controls increased the production of IL-1α (Days 1–5), IL-36 (Days 4–5), and CXCL8 (Days 1–5) (Supplemental Table 1). No changes were observed between acetone and no exposure controls for VEGF and EGF. To assess skin barrier function, the permeability assay was performed after 5 days of repeated triclosan exposure. Exposure to triclosan for 5 days did not significantly increase tissue permeability (Figure 6). Together, these data demonstrate that repeated exposure to triclosan on EpiDerm tissues alters the expression patterns of cytokines and growth factors in the absence of permeability alterations.

## Discussion

In the United States, over 32 million workers are predicted to have the potential for exposure to chemicals that can be absorbed through the skin and cause occupational skin diseases (NIOSH (National Institute of Occupational Safety and Health) 2021; BLS (Bureau of Labor Statistics) 2022). Investigations into skin exposures are needed, because these exposures can lead to immunotoxicity and allergic disease (NORA (National Occupational Research Agenda) 2019). The anti-microbial chemical triclosan is used in occupational settings that result in exposure to the skin and has been shown to increase the allergic response and production of pro-inflammatory cytokines in animal models. However, the direct impacts of triclosan exposure on skin cells and the skin barrier are not well understood.

In this study, the impact of a single or repeated exposure to triclosan on the skin barrier and keratinocyte response was investigated using the RHE, EpiDerm. This study showed that a single exposure of triclosan on EpiDerm tissues significantly increased the expression of genes involved in skin barrier function, including *FLG*, *FLG2*, and *KRT10* (Figure 1). However, *FLG*, *FLG2*, and *KRT10* gene expression were not significantly changed after the repeated exposure (Figure 4). These results partially align with animal studies that demonstrate that exposure to triclosan on SKH1 hairless mouse skin decreased *Flg2* and *Krt10* gene expression (Baur et al. 2021). While the importance of *FLG* in clinical atopic dermatitis has been well reported, investigations using *in vitro* models are conflicting. Previous studies have shown that knockdown of *FLG* in RHE alters tissue permeability (Mildner et al. 2010; Pendaries et al. 2014), but, in contrast, RHE constructed with keratinocytes with *FLG* loss-of-function mutations has not been shown to alter tissue permeability (Niehues et al. 2017). Although *FLG2* knock-down in RHE has not been shown to alter permeability, other markers of skin integrity including decreased abundance of urocanic acid, decreased loricrin expression, and disrupted processing of profilaggrin were identified (Pendaries et al. 2015). However, the role of *FLG2* in barrier integrity has been suggested by other studies. Knockdown of *FLG2* in a RHE model was shown to result in more fragile cornified envelopes, an essential component of skin barrier integrity (Albérola et al. 2019). Additionally, the impact of chemical exposure on *FLG* and *FLG2* has not been well investigated. A single exposure to triclosan on EpiDerm tissues also increased expression of *TJP1* and *OCLN* (Figure 1), barrier genes which contribute to the formation of tight junctions and regulate tissue permeability (Kirschner et al. 2013). Together, these results demonstrate that triclosan alters the expression of skin barrier genes *in vitro* and suggests that integrity of the barrier may be impacted due to changes in these genes. Additionally, exposure duration and timing may also be a contributing factor.

In this study, *TSLP* expression was significantly increased in EpiDerm tissues after 6 h of triclosan exposure (Figure 2(A)), but not after 5 days of repeated exposure (Figure 5(A)). Previously, dermal exposure to triclosan was shown to increase *Tslp* expression in mouse skin in T_H_2-response-prone BALB/c mice (Anderson et al. 2020). TSLP is produced by keratinocytes and is involved in T_H_2 immune responses (Hammad and Lambrecht 2015). Although TSLP stimulation of human keratinocytes was previously shown to decrease expression of *FLG* (Kim et al. 2015), differences between levels of TSLP added to cultured human keratinocytes (over 10 ng/ml) (Kim et al. 2015) compared to TSLP levels produced by EpiDerm in this study (less than 0.003 ng/ml) may account for the differential responses in *FLG*.

Exposure to triclosan also increased EpiDerm tissue *IL1A* gene expression (Figure 2(B)) and IL-1α protein production (Table 1) and repeated exposure to triclosan increased IL-1α production/release (Table 2) as early as 24 h post exposure. IL-1α is one of the first cytokines released following keratinocyte stimulation, leading to the production of additional pro-inflam-matory cytokines (Jiang et al. 2020). Additionally, IL-1α may be involved in repair of the skin barrier (Gutowska-Owsiak and Ogg 2013). Although the acetone vehicle increased IL-1α levels too, compared to no exposure controls, exposure to triclosan further increased the release of IL-1α when compared to acetone vehicle, demonstrating that exposure to triclosan impacts the release of pro-inflammatory cytokines. Taken together, these results demonstrate that triclosan exposure on EpiDerm tissues has a direct impact on the keratinocyte pro-inflammatory and allergic response.

A single exposure to triclosan on EpiDerm tissues increased *CXCL1*, *CXCL2*, *TNF*, and *CXCL8* gene expression (Figure 2(D–G)) suggesting a pro-inflammatory response. While CXCL8 was elevated at the transcript level, protein levels were not significantly changed. The most robust change in gene expression occurred at the 6-h timepoint, though protein levels were not evaluated at this timepoint. By 48 h, no changes were identified in gene expression, suggesting this finding might be a result of kinetics. An increased expression of *CXCL2* and *CXCL1* aligns with increased *Cxcl2* and *Cxcl1* gene expression in mouse skin following triclosan exposure (Weatherly et al. 2020). Additionally, in this EpiDerm model, TSLP protein production was below the limit of detection which could also be due to the lack of immune cells in this model, since mast cells, basophils, and dendritic cells have also been shown to be a source of TSLP (Tsilingiri et al. 2017). A single exposure to triclosan on EpiDerm tissues increased protein production of IL-36 and VEGF (Table 1) and repeated exposure to triclosan increased IL-36 and EGF protein levels (Table 2). Application of activated IL-36 on a 3 D skin model constructed with normal human epidermal keratinocytes has been shown to down-regulate genes involved in skin integrity (*FLG*, *FLG2*, *LOR*, *KRT10*) (Pfaff et al. 2017). Furthermore, EGF may regulate IL-36 (Buhl and Wenzel 2019). EGF receptor knockout mice were shown to have elevated levels of IL-36, suggesting that EGF signaling controls IL-36 levels (Franzke et al. 2012). Although VEGF was initially increased following triclosan exposure, production of VEGF decreased after repeated exposures, along with CXCL8. This suggests that pro-inflammatory effects occurred early-on but waned with time, potentially due to cytotoxicity.

One limitation of this study is the cytotoxicity that was observed following both a single and repeated exposure to triclosan (Figures 1(A) and 4(A)). Cytotoxicity has previously been shown in cultured mouse keratinocytes (Wu et al. 2015) and EpiDerm tissues (Marshall et al. 2015) following triclosan exposure. The observed cytotoxicity may be in part due to the use of acetone vehicle which was shown in the present study to increase the production of pro-inflammatory cytokines (e.g. IL-1α) compared to no exposure control (Supplemental Table 1). The cytotoxicity observed in the EpiDerm model may limit a full understanding of the effects of triclosan exposure and this limitation of the *in vitro* model should be considered in future investigations. In mice, exposure to triclosan on the skin has been shown to cause skin lesions, suggesting dermal toxicity (Fang et al. 2015). However, *in vitro*, cell regeneration and healing may be limited, unlike in the *in vivo* model. Surprisingly, repeated exposure to triclosan on EpiDerm tissues also decreased *S100A8* gene expression (Figure 5(E)). This differential change in an immune-related gene, compared to animal studies where dermal exposure to triclosan significantly increased *S100a8* gene expression (Marshall et al. 2017; Weatherly et al. 2020), may again be due to differences in cell populations, because no immune cells, such as neutrophils, are present in EpiDerm tissues, or due to cytotoxic effects. Taken together, changes in the expression patterns of cytokines and growth factors indicate changes in keratinocyte function following triclosan exposure in this model of RHE.

Disruptions in skin barrier genes and cytokines can impact skin barrier integrity. A single exposure to triclosan on EpiDerm tissues increased tissue permeability (Figure 3(A)), demonstrating a disruption in barrier integrity. Although studies investigating single genes involved in the skin barrier, such as *FLG2* (Pendaries et al. 2015), have not been shown to directly disrupt tissue permeability, the combination of barrier genes and cytokines changing in keratinocytes due to triclosan exposure may be responsible for the impact on tissue permeability. Previously, we have shown that exposure to triclosan on mouse skin disrupts barrier integrity (Baur et al. 2021), complimenting these *in vitro* findings on skin integrity. Interestingly, repeated (5-day) exposure to triclosan had no statistically significant impact on skin permeability nor expression of skin barrier genes. This result may be due to the overt cytotoxicity observed following repeated triclosan exposure. Additionally, the length of exposure (5 days) may account for differences observed, compared to 1 day of exposure. This highlights some of the limitations of the *in vitro* model and supports the need for additional kinetic related studies.

## Conclusions

The results of the present study showed that exposure to triclosan on RHE disrupts expression of skin barrier genes, increases barrier permeability, and alters keratinocyte function which is consistent with animal studies and further expands on previous observations by using a human skin model. Changes in keratinocyte function following exposure to chemicals may be a critical initiator in recruitment and signaling to immune cells, followed closely by disruption of the skin barrier, resulting in increased absorbance of chemicals and further exacerbation of the pro-inflammatory and allergic response. Studies investigating the response of exposure to chemicals, including anti-microbials, on the skin are critical for increased understanding of early changes in the skin and the initiation of sensitization or immune modulation.

## Supplementary Material

supplemental material

## Figures and Tables

**Table 1.** Cytokines and growth factors (pg/ml) released following 24 and 48 h of triclosan exposure on EpiDerm.

Exposure Duration Triclosan (w/v)	24h				48h			
	0%	0.05%	0.1%	0.2%	0%	0.05%	0.1%	0.2%
IL-1α	19.4 ± 2.2	29.3 ± 3.6	*39.7 ± 2.8	*54.0 ± 9.1	26.2 ± 11.1	32.4 ± 11.9	*61.0 ± 26.2	*71.8 ± 14.7
IL-36	49.5 ± 3.2	59.1 ± 3.3	*70.4 ± 10.7	*99.3 ± 6.0	78.7 ± 9.8	84.1 ± 5.8	*114.3 ± 9.8	*145.9 ± 19.3
CXCL8	72.7 ± 7.7	54.5 ± 1.5	59.9 ± 6.2	46.9 ± 5.6	100.4 ± 38.3	93.8 ± 29.0	115.7 ± 54.7	66.3 ± 25.8
VEGF	79.8 ± 20.6	*102.0 ± 20.9	*121.2 ± 18.4	*129.1 ± 9.6	112.8 ± 32.7	136.2 ± 27.0	*154.0 ± 27.8	142.7 ± 38.0
EGF	28.5 ± 21.1	31.0 ± 19.9	28.6 ± 19.0	31.7 ± 22.7	39.6 ± 3.8	44.1 ± 0.3	42.9 ± 6.0	43.4 ± 3.6

Numbers represent mean (± SEM) of two samples/group. *p < 0.05 vs. 0% triclosan.

**Table 2.** Cytokines and growth factors (pg/ml) released following 5 days of triclosan exposure on EpiDerm.

Cytokine	Triclosan (w/v)	1 Day	2 Days	3 Days	4 Days	5 Days
IL-1α	0%	74.3 ± 12.9	94.2 ± 33.6	136.8 ± 10.7	140.6 ±13.8	163.2 ± 29.8
	0.1%	*181.9 ± 98.0	167.9 ± 78.5	211.9 ± 9.7	200.2 ± 8.7	186.8 ± 27.4
	0.2%	*254.0 ± 119.8	*198.5 ± 74.5	249.9 ± 10.0	184.6 ± 21.9	154.3 ± 30.7
IL-36	0%	127.3 ± 80.8	74.8 ± 53.4	59.5 ± 30.7	51.6 ± 26.9	50.1 ± 21.4
	0.1%	240.1 ± 157.8	179.5 ± 107.2	*211.7 ± 38.0	*218.4 ± 23.3	*222.9 ± 42.0
	0.2%	*371.0 ± 243.0	*236.2 ± 126.7	*298.2 ± 45.3	*257.4 ± 45.7	*212.1 ± 50.8
CXCL8	0%	332.2 ± 188.3	230.7 ± 100.0	269.7 ± 20.8	246.9 ± 29.5	243.2 ± 30.0
	0.1%	372.9 ± 237.9	181.5 ± 93.4	127.0 ± 8.4	71.0 ± 4.1	*42.5 ± 3.0
	0.2%	361.0 ± 240.4	152.6 ± 88.4	88.6 ± 13.5	*43.7 ± 1.7	*27.2 ± 0.9
VEGF	0%	336.6 ± 115.9	274.0 ± 129.9	40.8	310.8 ± 45.5	326.8 ± 74.3
	0.1%	563.8 ± 203.9	456.0 ± 205.0	487.0 ± 110.5	302.4 ± 139.2	*186.5 ± 130.3
	0.2%	498.3 ± 224.1	312.9 ± 165.3	*178.9 ± 47.0	*68.5 ± 19.5	*38.9 ± 13.3
EGF	0%	21.2 ± 3.9	17.9 ± 6.8	22.6 ± 0.9	23.0 ± 1.3	21.4 ± 2.4
	0.1%	24.0 ± 9.1	22.7 ± 9.5	33.1 ± 0.1	*40.1 ± 1.6	*44.2 ± 3.2
	0.2%	25.2 ± 10.2	30.2 ± 11.4	*48.7 ± 3.5	*54.3 ± 7.1	*56.6 ± 10.8

Numbers represent mean (± SEM) of two samples/group. *p < 0.05 vs. 0%.
