# Supplementary material for: Exposure to the anti-microbial chemical triclosan disrupts keratinocyte function and skin integrity in a model of reconstructed human epidermis
Source: J Immunotoxicol. Author manuscript; Available in PMC 2023 Dec 1. (PMC10364087; doi:10.1080/1547691X.2022.2148781)
Supplement: supplemental material [file NIHMS1899701-supplement-supplemental_material.doc]

# Supplemental Figure legends

**Supplemental Figure 1.** Exposure to triclosan on EpiDerm tissues increased expression of additional select skin barrier genes. **(A)** RNA yield (ng/µl) following 6, 24, and 48 hr of 0% triclosan (acetone vehicle) or 0.05-0.2% triclosan. Bars represent mean (± SEM) of two samples/group. *p < 0.05 vs. 0% triclosan. Fold-change in gene expression compared to vehicle control of **(B)** *IVL*, **(C)** *LOR*, and **(D)** *CDH1* following 6, 24, and 48 hr of 0% triclosan (acetone vehicle) or 0.05-0.2% triclosan. Bars represent mean (± SEM) of two samples/group. *p < 0.05 vs. 0% triclosan.

**Supplemental Figure 2.** Expression of additional select skin barrier genes following repeated triclosan exposure on EpiDerm tissues. **(A)** RNA yield (ng/µl) following 5 days of 0% triclosan (acetone vehicle) or 0.1-0.2% triclosan. Bars represent mean (± SEM) of two samples/group. *p < 0.05 vs. 0% triclosan. Fold-change in gene expression compared to vehicle control of **(B)** *IVL*, **(C)** *LOR*, and **(D)** *CDH1* following 5 days of 0% triclosan (acetone vehicle) or 0.1-0.2% triclosan. Bars represent mean (± SEM) of two samples/group. *p < 0.05 vs. 0% triclosan.

**Supplemental Figure 1**


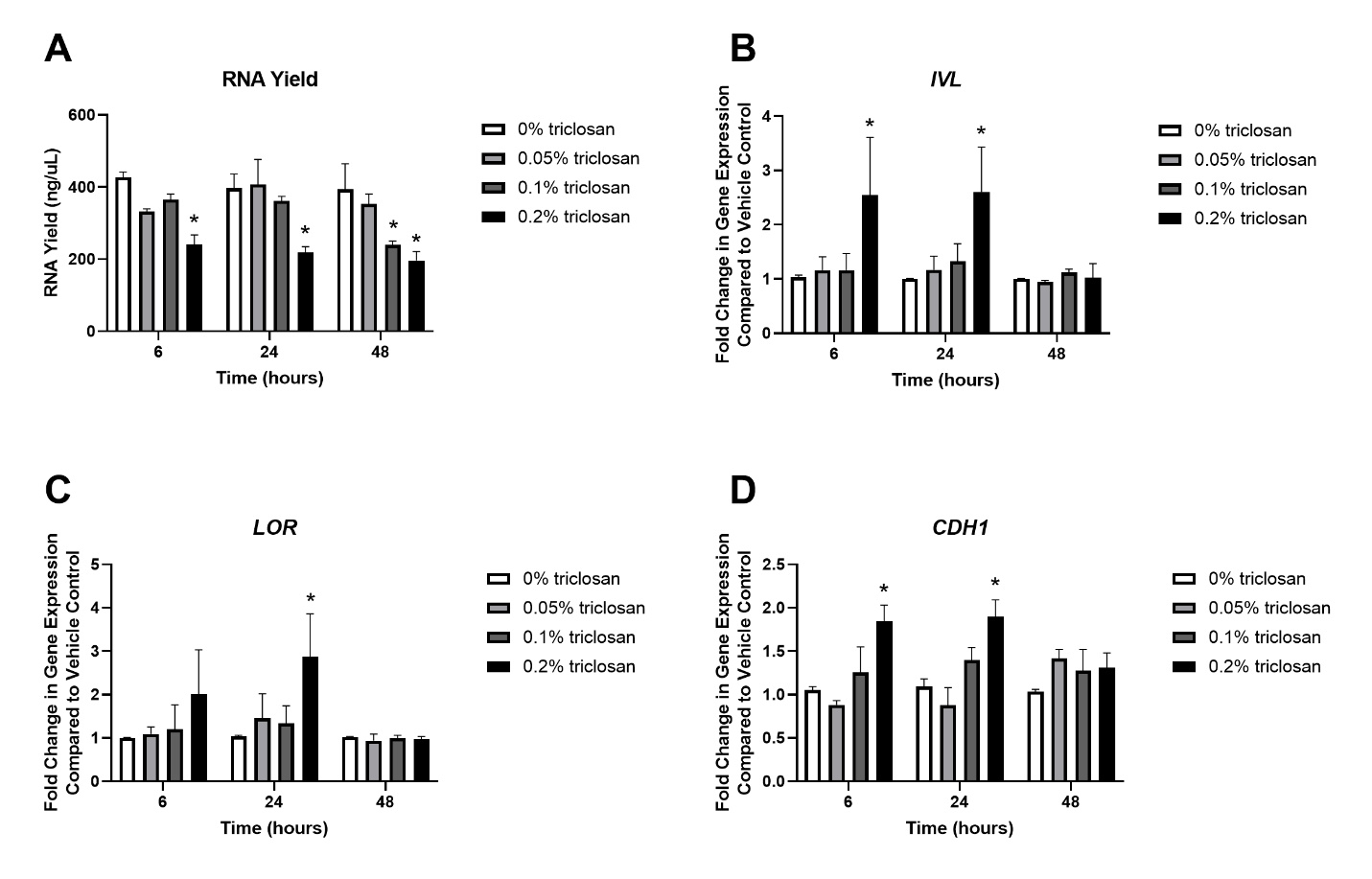


**Supplemental Figure 2**


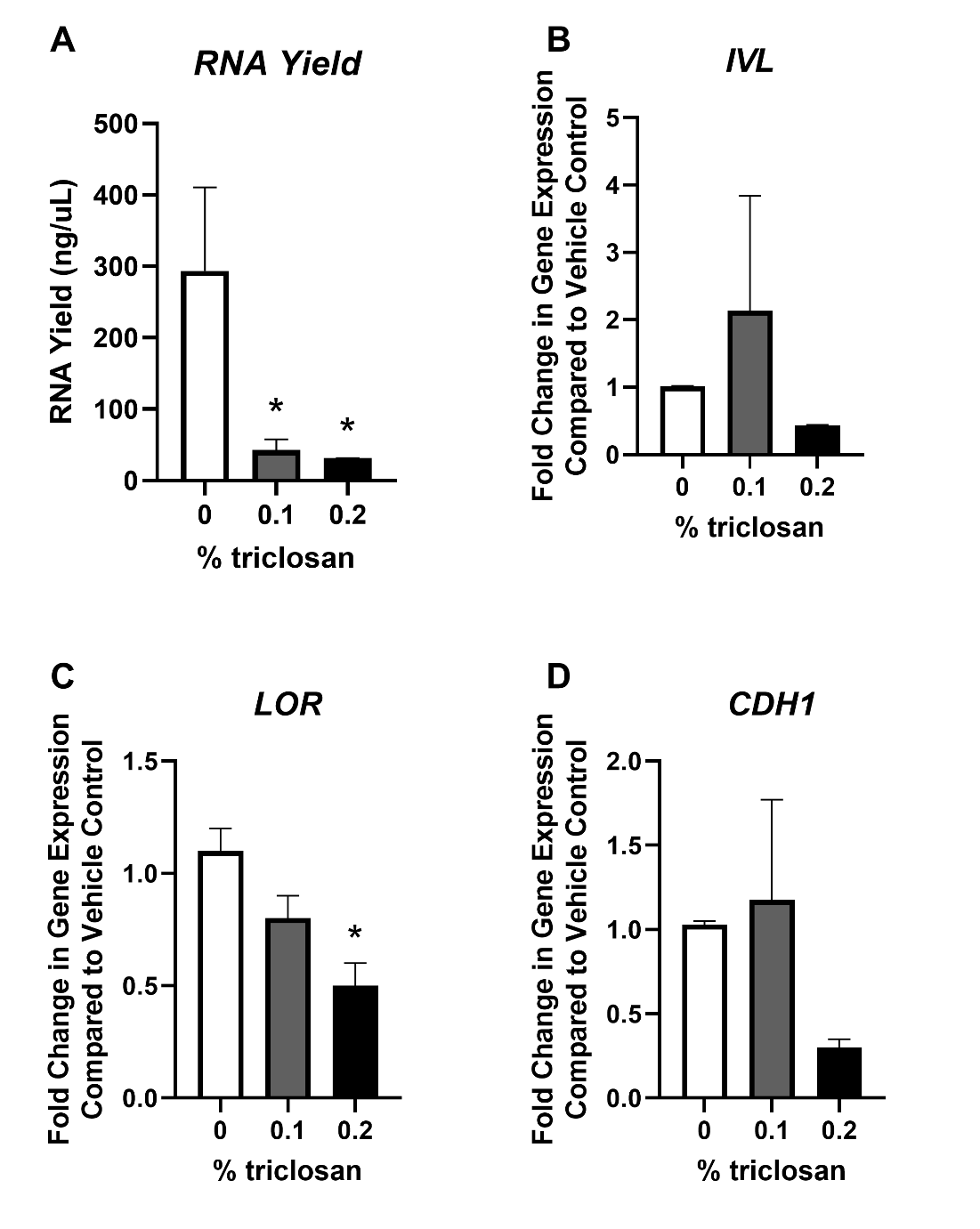


Supplemental Table 1

| **Cytokine** | **Exposure** | **1 day** | **2 days** | **3 days** | **4 days** | **5 days** |
| --- | --- | --- | --- | --- | --- | --- |
| **IL-1α** | No Exposure | 37.4 | 32.2 | 23.9 | 19.9 | 25.2 |
|  | Acetone | 87.3 | 127.7 | 147.4 | 154.3 | 192.9 |
| **IL-36** | No Exposure | 155.5 | 104.4 | 68.73 | 50.9 | 50.4 |
|  | Acetone | 208.1 | 128.3 | 90.20 | 78.5 | 71.4 |
| **CXCL8** | No Exposure | 331.4 | 184.2 | 129.1 | 102.7 | 104.5 |
|  | Acetone | 520.5 | 330.7 | 248.8 | 217.4 | 213.3 |
| **VEGF** | No Exposure | 425.5 | 383.2 | 358.0 | 313.3 | 362.2 |
|  | Acetone | 452.5 | 404.0 | 380.5 | 356.2 | 401.1 |
| **EGF** | No Exposure | 25.5 | 24.1 | 22.5 | 22.5 | 20.9 |
|  | Acetone | 25.0 | 24.7 | 23.5 | 24.3 | 23.8 |

Cytokines and growth factors (pg/ml) released after 5 days of acetone exposure on EpiDerm.
